# The leukotriene receptor antagonist montelukast as a potential therapeutic adjuvant in multiple sclerosis – a review

**DOI:** 10.3389/fphar.2024.1450493

**Published:** 2024-09-13

**Authors:** Frank Pietrantonio, Alex Serreqi, Horst Zerbe, Per Svenningsson, Ludwig Aigner

**Affiliations:** ^1^ IntelGenX Corp., Saint-Laurent, QC, Canada; ^2^ Department of Clinical Neuroscience, Neuro Svenningsson, Karolinska Universitetssjukhuset, Stockholm, Sweden; ^3^ Institute of Molecular Regenerative Medicine, Paracelsus Medical University, Salzburg, Austria

**Keywords:** regeneration, restoration, drug development, remyelination, neuroinflammation

## Abstract

Multiple Sclerosis (MS) is a multifactorial autoimmune disease of the central nervous system (CNS). It is characterized by a heightened activation of the immune system with ensuing inflammation, demyelination and neurodegeneration with consequences such as motor, sensory, cognitive, as well as autonomic dysfunctions. While a range of immune-modulatory drugs have shown certain efficacy in alleviating pathology and symptoms, none of the currently available therapeutics regenerates the damaged CNS to restore function. There is emerging evidence for leukotrienes and leukotriene receptors being involved in the various aspects of the MS pathology including neuroinflammation and de/remyelination. Moreover, leukotriene receptor antagonists such as the asthma drug montelukast diminish inflammation and promote regeneration/remyelination. Indeed, montelukast has successfully been tested in animal models of MS and a recent retrospective case-control study suggests that montelukast treatment reduces relapses in patients with MS. Therefore, we propose montelukast as a therapeutic adjuvant to the standard immune-modulatory drugs with the potential to reduce pathology and promote structural and functional restoration. Here, we review the current knowledge on MS, its pathology, and on the potential of leukotriene receptor antagonists as therapeutics for MS.

## Introduction

Multiple Sclerosis (MS) is a multifaceted degenerative disease of the central nervous system (CNS), which is typically characterized by a heightened activation of the immune system with ensuing inflammation, demyelination and neurodegeneration (Filippi et al., 2018). MS patients suffer from motor, sensory, autonomic, as well as cognitive dysfunctions (Chiaravalloti and DeLuca, 2008). Although the exact etiology remains nebulous and elusive, the disease appears to result from an association between genetic susceptibility and exposure to environmental risk factors stirring up demyelination and neurodegeneration (Raffel et al., 2016). Other contributing factors may include age, sex, diet, smoking, injuries and previous viral infections (Dighriri et al., 2023).

MS is an acquired idiopathic demyelinating autoimmune disease of the CNS, characterized by axonal loss, astrocytic scarring (gliosis) and progressive neurodegeneration (Mahad et al., 2015; Campbell and Mahad, 2018; Dendrou et al., 2015; Lassmann, 2019). Resultant tissue damage associated with the course of the disease stems from intricate and dynamic interactions among the immune system, glia (oligodendrocytes and their precursors, microglia, and astrocytes), and neurons (Reich et al., 2018). The associated damage due to the initiation and perpetuation of inflammatory mediators from the above-mentioned interplay eventually gives rise to focal plaques or lesions, the hallmarks of the disease. These lesions or plaques represent demyelinated white and gray matter of the brain and spinal cord indicating damage to and loss of the axonal myelin sheaths due to the ongoing oligodendrocyte apoptotic activity (Dendrou et al., 2015; Lassmann, 2019). Typically, these detectable plaques, observable by MRI, mainly appear in the white matter surrounding the ventricles, optic nerves and tracts, corpus callosum, cerebellar peduncles, long tracts and subpial area of the spinal cord and brainstem, as well as in the gray matter (Compston and Coles, 2008). Demyelination of the axonal myelin sheaths leads to various effects, including alterations in nodal saltatory conduction, slowed conduction velocity and a predisposition to conduction block (Davis et al., 2010).

To date, the majority of the US Food and Drug Administration (FDA) approved treatments for MS are either immunomodulatory or immunosuppressive in nature. These treatments have played a pivotal role in alleviating many associated symptoms stemming from the disease progression, while also offering valuable insight into new treatment modalities (Table 1). However, despite the welcomed presence of these immunomodulatory treatments, a notable gap remains: they do not reverse or boost remyelination, nor do they prevent disease progression or neuronal damage (Heβ et al., 2020; Melchor et al., 2019; Marques et al., 2022; Han et al., 2021).

**TABLE 1:** Drugs in use as therapeutics in MS and montelukast.

Drug	Tradename	Drug class/type	Target	Mode of Action
Interferon beta-1a	Avonex® Plegridy® Rebif®	Immunomodulator Interferon	T cells	Anti-inflammatory Anti-viral
Interferon beta-1b	Betaseron® Extavia®	Immunomodulator Interferon	T cells	Anti-inflammatory Anti-viral
Glatiramer acetate	Copaxone® Glatopa®	Immunomodulator	T cells	Anti-inflammatory
Natalizumab	Tysabri®	Humanized monoclonal antibody Immunosuppressive	α4-integrin	Anti-inflammatory prevents immune cells from crossing the blood-brain barrier
Alemtuzumab	Lemtrada®	Humanized monoclonal antibody Immunosuppressive	CD52	Anti-inflammatory B-and T-cell depletion
Cladribine	Mavenclad®	Nucleoside analogues Cytostatic Immunosuppressive	B- and T-cells	Anti-inflammatory B-and T-cell depletion
Ublituximab	Briumvi®	monoclonal antibody Immunomodulator	CD20	Anti-inflammatory B-cell depletion
Ocrelizumab	Ocrevus®	Immunosuppressive humanized monoclonal antibody	CD20	Anti-inflammatory B-cell depletion
Ofatumumab	Kesimpta®	Humanized monoclonal antibody Cytostatic	CD20	Anti-inflammatory B-cell depletion
Fingolimod	Gilenya®	Immunosuppressive Immunomodulator	sphingosine-1-phosphate (S1P) receptors 1, 3, 4 and 5	Anti-inflammatory Trapping immune cells in lymph nodes
Ozanimod	Zeposia®	Immunomodulator	S1P receptor 1 and 5	Anti-inflammatory Trapping immune cells in lymph nodes
Siponimod	Mayzent®	Immunomodulator	S1P receptor 1 and 5	Anti-inflammatory Trapping immune cells in lymph nodes
Ponesimod	Ponvory®	Immunomodulator	S1P receptor 1	Anti-inflammatory Trapping immune cells in lymph nodes
Mitoxantrone	Novantrone®	Antibiotic Cytostatic Immunosuppressive	type II topoisomerase	Anti-inflammatory
Teriflunomide	Aubagio®	Immunosuppressive	Dihydroorotate dehydrogenase	Anti-inflammatory *Neuroprotective*
Dimethyl fumarate	Tecfidera®	Immunomodulator	nuclear factor erythroid 2-related factor 2 (NRF2)	Anti-inflammatory Anti-oxidativ *Neuroprotective*
Monomethyl fumarate	Bafiertam®	Immunomodulator	NRF2	Anti-inflammatory Anti-oxidativ *Neuroprotective*
Diroximel fumarate	Vumerity®	Immunosuppressive Immunomodulator	NRF2	Anti-inflammatory Anti-oxidativ *Neuroprotective*
Montelukast	Singulair®	Leukotriene receptor antagonists	LTR, GPR17	Anti-inflammatory Pro-regenerative Pro-remyelinating

Table present an overview on currently available and approved drugs in MS., compared to montelukast, which has an anti-inflammatory as well as a pro-regenerative mode of action, the currently available MS, drugs are primarily immune-modulatory/anti-inflammatory. Cursive: possible mode of action, not proven.

## Leukotrienes and leukotriene receptors in MS

An overwhelming body of evidence from both nonclinical and clinical studies underscores the pivotal role of leukotrienes (LTs) in various physiological and pathological conditions. These highly active and potent lipid mediators synthesized from arachidonic acid via the 5-lipoxygenase (5-LOX) pathway have been extensively investigated. Elevated levels of LTs, including the cysteinyl leukotrienes (CysLTs) C_4_ (LTC_4_), LTD_4_, and LTE_4_, have been associated with specific age- and disease-related brain pathologies such as neuroinflammation, microglia/astroglia activation, neuronal damage, and increased blood-brain-barrier permeability (Michael et al., 2020a; Gelosa et al., 2017). In MS patients, levels of LTs in the CSF and urine are elevated as compared to control patients with non-inflammatory neurological disorders, in some cases approximately twice that of control patients (Neu et al., 2002; Haupts et al., 1992; Neu et al., 1992; Rosnowska et al., 1997). The enzyme 5-LOX plays a key role in the biosynthesis of pro-inflammatory LTs. Its expression is significantly increased in MS as well as in experimental autoimmune encephalomyelitis (EAE) mouse models of MS (Whitney et al., 2001). More specifically, gene microarray analysis has revealed an upregulation of the 5-LOX gene in brain lesions of patients with primary progressive MS (PPMS) and relapse remitting MS (RRMS) (Whitney et al., 2001; Arthur et al., 2008). In addition, immunohistochemistry analysis further corroborated these findings, highlighting predominant 5-LOX expression in macrophages within active and inactive MS lesions (Whitney et al., 2001). Furthermore, 5-LOX gene expression is elevated in peripheral blood cell samples from patients with RRMS during relapse and remission phases (Arthur et al., 2008).

The classical type 1 and type 2 CysLT receptors (CysLTR_1_ and CysLTR_2_) play an active role in most of the known CysLTs biological responses (Wang et al., 2020; Bäck et al., 2011; Bäck et al., 2014). Expression of LT receptors has been observed in multiple cell types of the CNS including astrocytes, neurons, endothelial cells and microglia spanning regions of the brain including the hippocampus, cortex, striatum and the substantia nigra (Peters-Golden and Henderson, 2007; Michael et al., 2020b; Marques et al., 2020; Wang et al., 2020; Gelosa et al., 2017). The CysLTR_1_ and CysLTR_2_ are upregulated in immune tissues, in serum, in cerebrospinal fluid (CSF), and in CNS tissue of EAE mice following disease onset and in MS patients (Wang et al., 2011; Han et al., 2021). Remarkably, infiltrating CD4^+^ cells, microglia and astrocytes within MS lesions express high levels of CysLTR_1_, mirroring similar observations as those in the EAE mice (Han et al., 2021).

Beside the classical receptors described above, other receptors involved in the leukotriene activation pathway include GPR99 (α-ketoglutarate receptor), P2Y_12_ (adenosine-diphosphate receptor) and GPR17 (uracil nucleotide P2Y-like receptor) (Marques et al., 2020). GPR17 was among the first leukotriene receptor with a putative role in MS. Initially identified as an orphan receptor, GPR17 turned out to be phylogenetically similar to CysLT receptors and to be activated and responsive to ligands such as uracil nucleotides (such as UDP, UDP-galactose and UDP-glucose) as well as to CysLTs (Ciana et al., 2006; Fumagalli et al., 2017; Lecca et al., 2020; Fumagalli et al., 2016; Dziedzic et al., 2020). Indeed, following trauma or disease such as acute ischemia and stroke, brain and spinal cord injuries, and demyelinating diseases such as MS, GPR17 expression is upregulated in neurons, microglia/macrophages, and most importantly in the context of de- and remyelination, in oligodendrocyte precursors (Ciana et al., 2006; Fumagalli et al., 2017; Lecca et al., 2020; Zhao B et al., 2018; Chen et al., 2009).

**FIGURE 1:**
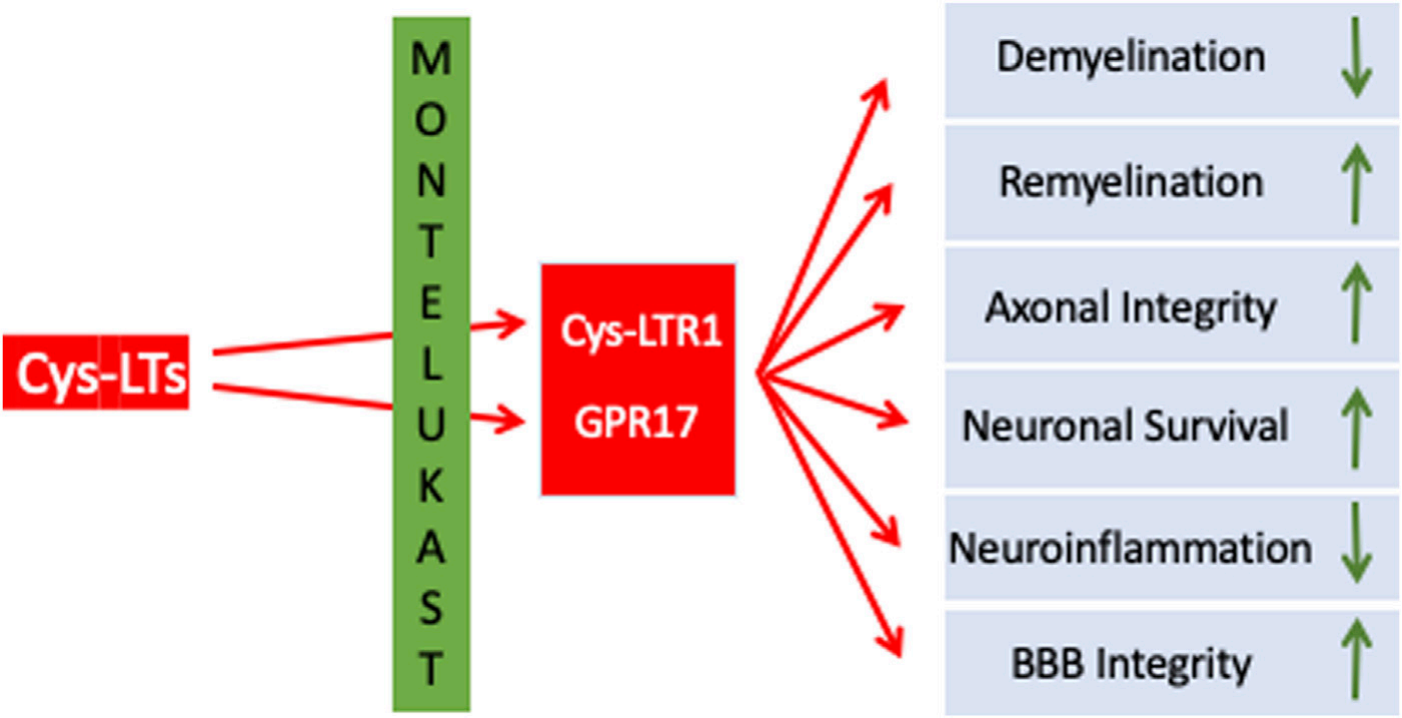
Summarizing scheme on the beneficial mode of actions of MTK in the context of MS.

GPR17 is abundantly expressed in the CNS, including the frontal cortex, striatum, brainstem, and medulla (Ceruti et al., 2010; Marucci et al., 2016). Within these brain regions GPR17 is expressed along the oligodendrocyte (OL) lineage. In remyelination, which is a constantly ongoing and physiological process in the adult CNS, OLs derive from oligodendroglial progenitors (OPCs) which are present throughout the CNS. In MS, particularly in more advanced stages of disease, remyelination is impaired, resulting in insufficient oligodendrogenesis and failure to remyelinate adequately. In consequence, changes in electrical impulse transmission, axonal damage and neurodegeneration progresses further without phases of symptomatic relief and recovery (Lecca et al., 2020; Dziedzic et al., 2020; Traiffort et al., 2020). In chronic MS lesions, proliferating OPCs were observed to have remained in situ without further differentiation into mature OLs (Kuhlmann et al., 2008). OPCs as well as OLs express GPR17 which is involved in regulating OPCs’ proliferation, differentiation, and migration. Actually, activation of GPR17 prevents OPC differentiation, proliferation and migration, i.e., GPR17 functions as a negative regulator for myelination. In plaques of MS patients GPR17 expression is higher compared to those from white matter from non-neurological donor samples and normal-appearing white matter (Chen et al., 2009). Most importantly, GPR17 overexpression in transgenic mice and GPR17 knockout mice demonstrated that GPR17 negatively regulates oligodendrocyte differentiation and myelination in vitro and in vivo in conditions of EAE. Therefore, inhibition of GPR17 seems to be a promising approach to promote remyelination. In summary, LTs as well as LTRs have been identified as therapeutic targets in MS. While the classical CysLTRs mediate neuroinflammation and related processes, GPR17 appears to be a roadblock in remyelination/regeneration. Targeting CysLTRs and GPR17 might simultaneously reduce inflammation and promote remyelination (Table 1 an Figure 1).

## Effects of leukotriene receptor antagonists in MS and in neurodegenerative diseases

Evidence for pharmacological leukotriene receptor blockade as a potential therapy in MS comes from studies using small molecule leukotriene receptor antagonists such as montelukast (MTK), pranlukast or zafirlukast. MTK is a selective and orally active leukotriene receptor antagonist that inhibits CysLTR_1_ as well as GPR17. It received FDA approval on February 1998 for several indications including the prophylaxis and chronic treatment of asthma, prevention of exercise-induced bronchoconstriction, and for the relief of symptoms of seasonal and perennial allergic rhinitis in adults and pediatric patients. Notably, MTK boasts an extensive safety and efficacy profile for asthma treatment, with over 25 years of clinical use and approval in more than 80 countries worldwide. The CysLTR_1_ antagonists zafirlukast and pranlukast act similarly to MTK, and have also FDA approval. Nevertheless, most of the studies exploring a potential effect in the CNS have been conducted using MTK.

With relevance to MS, MTK administration in mice reduced CysLTR_1_ mediated effects such as CNS infiltration of inflammatory cells such as Th1 and Th17, demyelination, BBB permeability, and clinical symptoms associated with EAE. Remarkably, MTK showed therapeutic efficacy even when given at the progressive stage of the disease in the EAE mice, suggesting its potential utility both as a prophylactic and as a therapeutic intervention (Wang et al., 2011). In another study, treatment of EAE mice with MTK resulted in a notable suppression or delay of EAE development both prophylactically and therapeutically, accompanied by inhibition of myelin loss (Han et al., 2021). Furthermore, inflammation in MTK treated EAE mice was suppressed as demonstrated by significant decreases in the percentages of inflammatory cells in the CNS, i.e., CD4^+^ IFN-γ+ (Th1), CD4^+^ IL-17+ (Th17) and granulocyte–macrophage colony-stimulating factor CD4^+^ GM-CSF+. This reduction corresponded with decreased concentrations of IFN-γ, IL-17 and GM-CSF cytokines. Th17 cells, critical in the pathogenesis of both EAE and MS, produce IFN-γ, IL-17 and GM-CSF cytokines, contributing to astrocytes and microglial activation, and subsequent cytokine and chemokine secretion. This cascade influences the recruitment of peripheral immune cells to lesion sites, exacerbating attacks on the CNS (Dendrou et al., 2015; Han et al., 2021; Zhang et al., 2018).

MTK as well as pranlukast also atangonize GPR17 and might reduce pathology and promote regeneration (Hennen et al., 2013; Ciana et al., 2006; Braune et al., 2021). For example, in an amyotrophic lateral sclerosis (ALS) mouse model, treatment with MTK blocked GPR17 and increased differentiation of OPCs from spinal cord samples (Bonfanti et al., 2020). We (Marschallinger et al., 2015) showed that MTK is able to reduce neuroinflammation, restore BBB integrity, increase neurogenesis and improve cognitive performance in aged rats. The stimulating effect on neurogenesis was apparently mediated by inhibition of GPR17. Furthermore, we (Michael et al., 2021) analyzed the effects of MTK on cognition, inflammation, and hippocampal gene expression in 5xFAD mice, a commonly used transgenic AD mouse model. MTK was highly effective in reducing the effects of neuroinflammation, through modulation of microglia phenotypes and the impairment of CD8^+^ T-cell infiltration. In addition, MTK was effective in improving cognitive outcomes in the AD mice. Importantly, the effects observed both in terms of neuroinflammation and cognition were dose-dependent with the higher dose being more effective. Likewise, MTK reduces alpha-synuclein load and restores memory in an animal model of dementia with Lewy Bodies (Marschallinger et al., 2020). Also, in an animal model of stroke, MTK reduced the size of ischemic lesions, improved axonal fiber connectivity and remyelination. It improved OPC recruitment and proliferation during the acute phase of damage, and increased their differentiation to mature oligodendrocytes at the chronic phase after stroke (Gelosa et al., 2019).

## Clinical development and repurposing of Montelukast as a therapeutic for MS and neurodegenerative diseases

We have recently developed and manufactured a novel mucoadhesive montelukast film for buccal application (Montelukast VersaFilm^®^) (Michael et al., 2020a). The MTK film is based on a versatile drug delivery platform technology that enables the development of oral thin films exhibiting improved product performance, such as rapid disintegration of the delivery vehicle without the need for water, fast buccal or sublingual absorption, the potential for faster onset and/or higher bioavailability, ease of administration for patients with swallowing difficulties, pleasant taste, and individual packaging for improved safety and convenience. MTK VersaFilm^®^ was initially developed as an oral mucoadhesive film to circumvent the limitations of MTK in its tablet form, such as inconsistent solubility, uptake, and bioavailability. Formulating MTK as a buccally applied film is advantageous with respect to its ease of use, especially for patients suffering from dysphagia, such as elderly patients, patients with dementia, and patients that require intubation or ventilation. For clinical testing, we developed and manufactured two dosage strengths, a 10-mg and a 30-mg film. The film is required to be placed on the inner cheek mucosa and then allowed to adhere for several seconds; complete dissolution of the film occurs in less than 5 min.

A Phase 1 and Phase 2a study was conducted with the MTK VersaFilm^®^ with either of the two dosages a 10-mg and a 30-mg film (Michael et al., 2020a). The Phase 1 bioavailability study in healthy volunteers compared a 10-mg tablet formulation to the 10-mg buccal film (Michael et al., 2020b). The currently ongoing Phase 2a study compares the effects of several daily doses of MTK (10-mg and 60-mg) to placebo in patients with mild to moderate Alzheimer’s disease (NCT03402503). Phase 1a study results established that the MTK film demonstrated a significantly improved bioavailability compared to the marketed tablet. For equivalent drug loadings (10 mg), MTK mucoadhesive film exhibited approximately 50% better bioavailability compared to MTK tablets. As part of the study, we also conducted a quantitative analysis of the MTK levels in the CSF and found measurable amounts of MTK present in the CSF at the 3.0- and 7.0-h time points after test drug administration (Michael et al., 2020b).

The purpose of the Phase 2a “Proof of Concept” study is to evaluate the safety and effectiveness of the Montelukast VersaFilm^®^ in combination with current established “Standard of Care” pharmacotherapy (acetylcholinesterase inhibitors - AChEIs) in patients with mild to moderate AD-associated dementia. The Phase 2a “Proof of Concept” study, ending in Q2 2024, will evaluate the effect on cognition and functional daily living in patients with mild to moderate AD dementia treated for 26 weeks with MTK VersaFilm^®^ along with AChEIs versus patients treated with placebo and AChEIs. The Montelukast VersaFilm^®^ is currently also being evaluated in a Swedish Phase 2 Parkinson’s disease (PD) study (MONTPARK - EudraCT Number: 2023–504278–39–00). The overall purpose of the study is to investigate the safety and efficacy of high oral doses of MTK on PD progression in the early to moderate phase of the disease. The study design is a randomized, double-blind, placebo-controlled, parallel arm, multicenter trial to examine the safety and efficacy of buccal films of MTK 30-mg taken twice daily compared to placebo film taken twice daily. Evaluable patients on the study will be treated for 18 months with either MTK or placebo and then undergo a 3-month washout period. Additional evaluations to be conducted as part of the study are to include evaluation of motor and non-motor functions, changes in dopaminergic treatment, safety, and plasma and CSF levels of inflammatory, leukotriene and neurodegeneration markers as well as MTK. Most importantly in the context of MS, a recent retrospective case-control study suggests that MTK reduces relapses in patients with MS (Manuel et al., 2024). This study used two large datasets from a total of 118,642 people, with 691 of them being adherent on MTK. Compared to the controls, MTK users showed a 58.3% reduction in the annualized relapse rate in one cohort, and a 13.4% reduction in the other cohort. This real-world evidence suggests that MTK reduced MS relapses, arguing for future clinical testing of the efficacy of MTK in MS patients in a randomized placebo-controlled trial.

## Summary and conclusion

MS, one of the many neurodegenerative diseases afflicting humans, is a complex phenomenon for which many of the underlying mechanisms and players have not been fully elucidated. Recommendations to date are to initiate early treatment of the MS patient in order to maximize the efficacy of the currently available therapies (Fumagalli et al., 2016). Most of these current available FDA-approved therapies for MS consist of immunomodulating and immunosuppressive approaches acting against the inflammatory and immune-mediated components of the disease (Melchor et al., 2019). Although many of these approved treatments shorten the duration of relapse and delay progression to secondary phase of MS, what is suboptimal or predominately lacking is the ability to promote CNS repair, i.e., remyelination (Melchor et al., 2019). Immunomodulation and immunosuppression cannot be solely counted on as an effective means to reverse or prevent remyelination failure to prevent the progression of MS to a state of permanent disability (Maciak et al., 2023). Remyelination is key to preventing progressive axonal injury and reducing long-term disability in the MS patients. OPCs are key to the remyelination process and an understanding of their involvement in all the phases of remyelination, i.e., OPC migration and proliferation, differentiation, OL maturation and OL survival are to be considered (Melchor et al., 2019).

Although a huge body of research is currently investigating various promising approaches to enhance remyelination in MS such as: replacement therapies, stem cell therapy, gene therapy, drugs stimulating growth factor production, immunomodulatory therapies and small-molecule therapies targeting specific signaling pathways that can promote remyelination, many of these strategies are far from immediate use in the clinical setting. The use of MTK warrants its inclusion in the current MS armamentarium given its inherent properties through its antagonistic effect on CysLT- and GPR17-receptors, which play a key influential role on OPC and microglia. MTK has been used for close to 25 years and its safety profile database in humans is extensive and well documented. Use of a buccal film of MTK, such as the one developed by us, provides additional advantages over the tablet formulation in terms of ease of use and greater bioavailability of up to 50% greater than that of the tablet formulation. An additional positive feature of utilizing a MTK buccal film is its use in MS patients with dysphagia. Dysphagia is a major disorder in MS patients and is defined as the difficulty in swallowing function. Several systematic reviews and meta-analyses have shown the prevalence of dysphagia in MS patients to be approximately 45% (Ansari et al., 2020; Mirmosayyeb et al., 2023), so the use of a mucoadhesive buccally applied film would be advantageous in this MS patient population. Lastly, future clinical studies are warranted to further evaluate the effect of a buccally applied MTK film in patients afflicted with MS as an adjuvant to currently approved therapy regimens. Although current therapies provide a mitigation strategy in terms of shortening the duration of relapse and delaying progression of the disease, most are suboptimal in their ability to promote CNS repair, i.e., remyelination; the use of MTK should be considered as part of the treatment regimen so as to delay or prevent progressive axonal injury and reduce long-term disability in the MS patient (Figure 1).
